# Do Physicians Respond to Web-Based Patient Ratings? An Analysis of Physicians’ Responses to More Than One Million Web-Based Ratings Over a Six-Year Period

**DOI:** 10.2196/jmir.7538

**Published:** 2017-07-26

**Authors:** Martin Emmert, Lisa Sauter, Lisa Jablonski, Uwe Sander, Fatemeh Taheri-Zadeh

**Affiliations:** ^1^ Institute of Management, School of Business and Economics Health Services Management Friedrich-Alexander-University Erlangen-Nuremberg Nuremberg Germany; ^2^ Media, Information and Design Department of Information and Communication University of Applied Sciences and Arts Hannover Germany

**Keywords:** Internet, online ratings, doctor-patient communication, public reporting, transparency

## Abstract

**Background:**

Physician-rating websites (PRWs) may lead to quality improvements in case they enable and establish a peer-to-peer communication between patients and physicians. Yet, we know little about whether and how physicians respond on the Web to patient ratings.

**Objective:**

The objective of this study was to describe trends in physicians’ Web-based responses to patient ratings over time, to identify what physician characteristics influence Web-based responses, and to examine the topics physicians are likely to respond to.

**Methods:**

We analyzed physician responses to more than 1 million patient ratings displayed on the German PRW, jameda, from 2010 to 2015. Quantitative analysis contained chi-square analyses and the Mann-Whitney *U* test. Quantitative content techniques were applied to determine the topics physicians respond to based on a randomly selected sample of 600 Web-based ratings and corresponding physician responses.

**Results:**

Overall, physicians responded to 1.58% (16,640/1,052,347) of all Web-based ratings, with an increasing trend over time from 0.70% (157/22,355) in 2010 to 1.88% (6377/339,919) in 2015. Web-based ratings that were responded to had significantly worse rating results than ratings that were not responded to (2.15 vs 1.74, *P*<.001). Physicians who respond on the Web to patient ratings differ significantly from nonresponders regarding several characteristics such as gender and patient recommendation results (*P*<.001 each). Regarding scaled-survey rating elements, physicians were most likely to respond to the waiting time within the practice (19.4%, 99/509) and the time spent with the patient (18.3%, 110/600). Almost one-third of topics in narrative comments were answered by the physicians (30.66%, 382/1246).

**Conclusions:**

So far, only a minority of physicians have taken the chance to respond on the Web to patient ratings. This is likely because of (1) the low awareness of PRWs among physicians, (2) the fact that only a few PRWs enable physicians to respond on the Web to patient ratings, and (3) the lack of an active moderator to establish peer-to-peer communication. PRW providers should foster more frequent communication between the patient and the physician and encourage physicians to respond on the Web to patient ratings. Further research is needed to learn more about the motivation of physicians to respond or not respond to Web-based patient ratings.

## Introduction

Over the last decade, physician-rating websites (PRWs) have become a popular tool to create more transparency about the quality of doctors in the United States, Germany, England, the Netherlands, Australia, Norway, Canada, and other industrialized countries [1-5]. PRWs are designed similarly to websites in other areas such as travel (eg, TripAdvisor and HRS), shopping (eg, Amazon), and restaurants (eg, Zagat). Besides the possibility of searching on the Web for physicians, patients can scan other patients’ reviews and also rate the received treatment. On PRWs, patients usually obtain structural information about a doctor’s office and results from Web-based patient satisfaction surveys [6]. Regarding the popularity of PRWs, a recently published article reported that 65% of US consumers are aware of PRWs, and 36% have gone on the Web to seek ratings or reviews about physicians [7]. These numbers are similar to those from Germany [8,9] and exceed those from other countries such as England [3]. Further surveys have shown that 1 out of 20 Internet users in the United States, and 1 out of 9 Internet users in Germany, have already rated a physician on the Web [7,9].

Much of what is known about PRWs is related to the level of awareness and usage among patients [3,7,9,10], the number and distribution of available Web-based ratings [5,11-13], ethical principles [14], underlying satisfaction survey instruments [15], the content of narrative review comments about physicians [16-18], the type of publicly reported quality information [6], pros and cons of PRWs in general [19], the association of Web-based ratings with clinical measurements of care [1,5,20-23], as well as the impact of Web-based ratings on patient care [4,24]. So far, less research has focused on the perspective of doctors who are being rated on PRWs [25]. What we have learned so far is that general practitioners in the United Kingdom had reservations and concerns about being rated on the Web. They mostly question the validity, usability, and transparency of Web-based ratings, as well as the resulting impact of Web-based ratings on them, their professional practice, and their relationship with their patients [25]. Besides, a study from Germany has demonstrated that Web-based patient ratings have an impact on the behavior of physicians and may have the potential to improve patient care. Here, more than half of the physicians surveyed (54.66%) used Web-based ratings to determine measures to improve patient care [4]. The most widely implemented quality measures were related to communication with patients (28.77%), the appointment scheduling process (23.60%), and office workflow (21.23%). However, we know little about whether and how physicians respond on the Web to patient ratings on PRWs. Learning more about those physicians who respond on the Web to patient ratings might also be beneficial if we want to further increase the usage of PRWs [25].

It thus seems important to gain a scientific understanding of whether and how physicians respond on the Web to patient ratings on PRWs and about the characteristics of those physicians who respond on the Web to patient ratings. In this context, this paper adds to the literature by presenting an analysis of all physician responses to Web-based patient ratings on the most popular German PRW, jameda, from 2010 to 2015. The following paper is divided into two parts. Part I contains results of quantitative analysis (1) to describe trends in physicians’ Web-based responses to patient ratings over time, and (2) to compare physicians who respond on the Web to patient ratings with nonresponders. In the second part (Part II), we used quantitative content analysis to evaluate a randomly selected sample of 600 Web-based ratings and corresponding physician responses from 2015 in detail. Based on those findings, we determined physician responses according to the topic and result of the Web-based patient rating.

## Methods

### Design and Data Source

**Figure 1:**
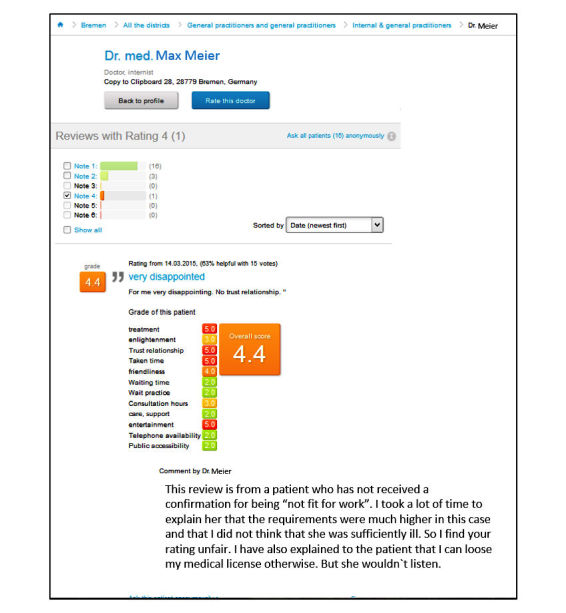
An example of a physician response to Web-based rating of a less-satisfied patient on jameda.

This paper presents an analysis of both patient ratings and physician responses, as well as physician responder characteristics displayed on the most popular German PRW, jameda, from 2010 to 2015. The mandatory rating system on jameda consists of 5 questions that have to be rated according to the grading system in German schools on a 1-6 scale (1=very good, 2=good, 3=satisfactory, 4=fair, 5=deficient, and 6=insufficient) [13]. These relate to (Q1) satisfaction with the treatment offered by the physician, (Q2) education about the illness and treatment, (Q3) the relationship of trust with the physician, (Q4) the time the physician spent on the patient’s concerns, and (Q5) the friendliness of the physician. A mean score (“overall performance”) is calculated afterwards, based on the results of these 5 questions. Beyond that, a narrative commentary must be given, and several optional questions are available for answering. Figure 1 displays an example of one physician response to a Web-based rating of a less-satisfied patient.

Data from the German PRW, jameda, from 2010 to 2015 were analyzed and contained slightly more than 1 million Web-based ratings and corresponding physician responses. The information included age, gender, and health insurance status of the patient, as well as the results of the physician ratings. Regarding the physician-related available data, the information included medical specialty and gender of the physician, the narrative response to the Web-based rating, the physicians’ overall rating, the membership status (ie, whether any service products are booked that contain different tools; eg, to modify or highlight a physicians’ profile [4]), the number of likes and site visits, as well as the percentage of how many patients would recommend the physician.

In our study, we analyzed both quantitative as well as qualitative data [26].

### Part I: Quantitative Analysis of Physicians’ Web-Based Responses

Regarding quantitative analysis, we performed comparisons between two groups by using a chi-square test (two-sided) for categorical variables and a Mann-Whitney *U* test for continuous nonparametric variables. (The Shapiro-Wilk test was applied to examine the normality of the data distribution.) In addition, we used the Phi coefficient to calculate the effect size for categorical variables and the formula by Fritz et al [27] for continuous nonparametric variables. All statistical analyses were conducted using SPSS version 22.0 (IBM Corp). Interrater agreement between the 2 raters was assessed using Cohen kappa coefficient (weighted). Differences were considered to be significant if *P*<.05 and highly significant if *P*<.001.

### Part II: Quantitative Content Analysis to Evaluate Physician Responses According to the Topic and Result of the Web-Based Patient Rating

Besides this, we used quantitative content analysis to determine the topics discussed in narrative patient comments [28] and topics physicians are most likely to respond to [29,30] based on previous evidence [16]. For this purpose, we analyzed a randomly selected sample of 600 Web-based ratings and corresponding physician responses from 2015. To assess differences between the 6 rating scores, we stratified the sample by rating score and collected 100 Web-based ratings and corresponding responses of each rating score. We applied an iterative process of both deductive and inductive steps for developing an all-embracing and disjunctive categorization framework that enabled us to capture the topics mentioned within the narrative comments and the physician responses. As a starting point, we used the categorization framework developed by Emmert and colleagues that distinguished between three main categories (ie, physician, office staff, and practice) and 50 subcategories (eg, patient involvement, communication, friendliness and caring attitude, information, and advice) [16]. This framework was extended in an iterative process; that is, new categories were added until a saturation of topics had been reached [31]. The final framework was pretested for 25 randomly selected pairs of narrative comments. Two of the authors independently carried out the assessment (n=450 for both coders, interrater agreement: 0.799; 95% CI 0.772-0.825).

## Results

### Part I: Quantitative Analysis of Physicians’ Web-Based Responses

In the following, we (1) describe trends in physicians’ Web-based responses to patient ratings over time and (2) compare physicians who respond on the Web to patient ratings with nonresponders. Table 1 shows the number of patient ratings and Web-based physician responses on the German PRW, jameda, from 2010 to 2015. Over the 6-year study period, slightly more than 1 million Web-based ratings were left for 142,948 rated physicians. The mean number of Web-based ratings per rated physician was calculated to be 7.36 (standard deviation [SD]=11.87; range 461) with a mean rating of 1.75 (SD=1.45; range 5). In total, 16,640 Web-based physician responses were left by 4187 physicians; in other words, physicians responded to 1.58% (16,640/1,052,347) of all Web-based ratings. Thereby, the percentage of Web-based ratings being responded to increased constantly over time from 0.70% (157/22,355) in 2010 to 1.88% (6377/339,919) in 2015. When regarding only those physicians who respond on the Web to patients’ reviews, the mean number of Web-based responses was 3.97 responses per physician (SD=9.64; range 241). The mean rating of responded Web-based ratings was 2.15 (SD 1.66) and significantly worse than of nonresponded Web-based ratings (mean rating=1.74, SD=1.45; *P*<.001). In absolute terms, most responses were given to answer to more favorable ratings; that is, 69.53% (11,571/16,640) of all responses were related to favorable comments, 14.34% (2387/16,640) to neutral comments, and 16.12% (2682/16,640) to negative comments, respectively. In relative terms, most responses were related to ratings in the middle range of the rating scale (3.54%, 962/27,167 for satisfactory and 3.57%, 1425/39,875 for fair overall ratings, respectively) but not to the most or least favorable ratings.

**Table 1:** An overview of the number of patient ratings and physician responses on jameda from 2010 to 2015.

Overview of patient ratings and physician responses	Year	Overall rating result^a^							Overall
		1	2	3	4	5	6		
**Web-based ratings, n**									
	2010	16,939	1817	619	877	1339	764	22,355	
	2011	39,591	3715	1455	1962	2890	1654	51,267	
	2012	103,342	9582	3405	4841	7294	5725	134,189	
	2013	159,250	15,212	5913	8426	13,367	11,058	213,226	
	2014	218,520	20,160	7660	11,465	18,643	14,943	291,391	
	2015	259,032	22,079	8115	12,304	20,702	17,687	339,919	
	Total	796,674	72,565	27,167	39,875	64,235	51,831	1,052,347	
**Physician responses, n (%)**									
	2010	87 (0.51)	10 (0.55)	17 (2.75)	14 (1.60)	21 (1.57)	8 (1.05)	157 (0.70)	
	2011	200 (0.51)	23 (0.62)	49 (3.37)	40 (2.04)	43 (1.49)	23 (1.39)	378 (0.74)	
	2012	809 (0.78)	83 (0.87)	80 (2.35)	110 (2.27)	166 (2.28)	77 (1.34)	1325 (0.99)	
	2013	1979 (1.24)	193 (1.27)	217 (3.67)	311 (3.69)	376 (2.81)	202 (1.83)	3278 (1.54)	
	2014	3327 (1.52)	294 (1.46)	276 (3.60)	434 (3.79)	526 (2.82)	268 (1.79)	5125 (1.76)	
	2015	4176 (1.61)	390 (1.77)	323 (3.98)	516 (4.19)	645 (3.12)	327 (1.85)	6377 (1.88)	
	Total	10,578 (1.33)	993 (1.37)	962 (3.54)	1425 (3.57)	1777 (2.77)	905 (1.75)	16,640 (1.58)	

^a^German school-based rating system (1=very good, 2=good, 3=satisfactory, 4=fair, 5=deficient, and 6=insufficient).

**Table 2:** A comparison of the responders and nonresponders of Web-based ratings on physician-rating websites (PRWs).

Characteristics	Responder (N=4187)	Nonresponder (N=138,761)	*P*-value^a,b^	Effect size
Gender (% female)	1035 (24.72)	51,615 (37.20)	<.001^a^	0.0431
Booked service package (% premium member)^c^	1652 (39.46)	5408 (3.90)	<.001^b^	0.2735
Web-based encounter scheduling tool (in %)^d^	332 (7.93)	853 (0.61)	<.001^a^	0.1325
Number of likes, mean (SD^e^)	98.8 (261.3)	25.5 (46.9)	<001^b^	0.1850
Site visits (Web-based profile on jameda), mean (SD)	17,789.1 (28,979.7)	5297.1 (7,214.9)	<.001^b^	0.1699
Recommended by patients, mean (SD^e^)	82.07 (17.10)	65.95 (34.97)	<.001^b^	0.0517
Overall Web-based rating (1-6 scale), mean (SD)	1.33 (0.47)	1.72 (0.98)	<.001^b^	0.0328

^a^Ch-square test (df=1 each).

^b^Mann-Whitney *U* test (Note: *P* values are adjusted for type 1 error by using the Holm-Bonferroni method).

^c^Service products contain different tools; for example to modify or highlight a physicians’ profile [1]).

^d^Web-based encounter scheduling tools allow to book an appointment on the Web.

^e^SD: standard deviation.

As shown in Table 2, physicians who responded on the Web to patient ratings (2.93%, 4187/142,948) differ significantly from nonresponders (97.07%, 138,761/142,948) in several aspects; they could be shown to be less likely to be female (mean=24.72% vs mean=37.20%), are more likely to have booked both one premium package (mean=39.46% vs mean=3.90%) and a Web-based encounter scheduling tool on jameda (mean=7.93% vs mean=0.61%), have a higher number of likes (mean=98.8, SD=261.3 vs mean=25.5, SD=46.9) and site visits on jameda (mean=17,789, SD=28,980 vs mean=5297, SD=7215), as well as both better patient recommendation results (mean=82.07; SD=17.10 vs mean=65.95; SD=34.97) and overall Web-based ratings (mean=1.33, SD=0.47 vs mean=1.72, SD=0.98; *P*<.001 each). As presented, the effect size was small and ranged between 0.0328 and 0.2735, respectively.

### Part II: Quantitative Content Analysis to Evaluate Physician Responses According to the Topic and Result of the Web-Based Patient Rating

Table 3 presents the number of patient ratings and physician responses to scaled-survey rating elements and narrative commentary, according to the topic and overall result of the patient rating, for a randomly selected sample of 600 Web-based ratings from 2015, which were equally distributed among the six overall rating result categories (ie, 100 ratings each). To leave a rating, patients had to rate numbers 1-5, whereas answering numbers 6-22 was optional. As shown for the scaled-survey mandatory rating topics (1-5), all 600 patients rated the friendliness and caring attitude of the physician. In addition, 268 patients described their experience of the friendliness and caring attitude of the physician in more detail using the narrative commentary. Here, every tenth physician (10.2%, 61/600) responded on the Web to this special aspect of the patient rating. The distribution of those 61 responses demonstrates that physicians were more likely to respond to lower patient ratings. For example, whereas approximately 20% of comments about the friendliness and caring attitude in negative ratings were responded to, this holds true only for 2% in very good ratings. In relative terms, most responses were given in answer to patient comments about the time spent with the patient (18.3%, 110/600). Again, physician responses were more likely for low ratings; for example, 28% of all 110 responses were given to ratings with an “insufficient” overall rating result.

With respect to the scaled-survey optional rating topics (6-22), the response rate varies between 0.4% (1/248) for patient ratings about additional treatment options and 19.4% for ratings concerning the waiting time within the practice (99/509). Regarding the latter, 498 patients used the scaled-survey rating system, and 142 patients provided additional information in the narrative commentary. As shown above, responses were more likely for lower ratings.

**Table 3:** An overview of the number of patient ratings and physician responses on jameda according to the topic of the rating for a randomly selected sample of 600 Web-based ratings (2015), equally distributed among the six overall rating result categories (ie, 100 ratings each).

Rating elements		Category	Topic	Patient ratings overall (Scaled survey ratings or narrative comments)	Physician responses, n (%)	Physician response rate per overall rating result (%)					
						Very good	Good	Satisfactory	Fair	Deficient	Insufficient
**Scaled-survey mandatory rating elements^a^**											
	1	Physician	Friendliness and caring attitude	600 (600/268)	61 (10.2)	2 (2.0)	4 (4.0)	12 (12.0)	5 (5.0)	17 (17.0)	21 (21.0)
	2	Physician	Satisfaction with treatment	600 (600/224)	95 (15.8)	6 (6.0)	11 (11.0)	18 (18.0)	17 (17.0)	21 (21.0)	22 (22.0)
	3	Physician	Time spent with the patient	600 (600/195)	110 (18.3)	9 (9.0)	19 (19.0)	20 (20.0)	13 (13.0)	21 (21.0)	28 (28.0)
	4	Physician	Information and advice	600 (600/126)	73 (12.2)	6 (6.0)	6 (6.0)	15 (15.0)	8 (8.0)	17 (17.0)	21 (21.0)
	5	Physician	Trust	600 (600/46)	31 (5.2)	4 (4.0)	4 (4.0)	3 (3.0)	10 (10.0)	6 (6.0)	4 (4.0)
**Scaled-survey optional rating elements**											
	6	Physician	Availability (eg, by telephone and email)	418 (417/10)	5 (1.2)	1 (1.1)	1 (1.2)	1 (1.4)	1 (1.6)	1 (1.6)	0 (0.0)
	7	Physician	Additional treatment options	275 (246/60)	39 (14.2)	2 (4.3)	6 (12.2)	12 (22.6)	4 (11.1)	8 (16.3)	7 (16.7)
	8	Physician	Child-friendliness	207 (203/12)	8 (3.9)	0 (0.0)	2 (5.3)	0 (0.0)	2 (6.1)	2 (7.1)	2 (6.3)
	9	Office staff	Service or assistance	466 (463/38)	8 (1.7)	3 (3.3)	1 (1.2)	2 (2.4)	0 (0.0)	1 (1.3)	1 (1.4)
	10	Office staff	Availability (eg, by telephone and email)	417 (417/1)	2 (0.5)	1 (1.1)	1 (1.2)	0 (0.0)	0 (0.0)	0 (0.0)	0 (0.0)
	11	Office staff	Additional treatment options	248 (246/3)	1 (0.4)	0 (0.0)	0 (0.0)	0 (0.0)	1 (3.1)	0 (0.0)	0 (0.0)
	12	Office staff	Child-friendliness	203 (203/3)	2 (1.0)	0 (0.0)	0 (0.0)	1 (2.6)	0 (0.0)	1 (3.6)	0 (0.0)
	13	Practice	Waiting time within the practice	509 (498/142)	99 (19.4)	6 (6.5)	25 (27.5)	13 (15.1)	19 (22.9)	21 (25.9)	15 (19.7)
	14	Practice	Waiting time to get an appointment	500 (490/86)	51 (10.2)	3 (3.2)	12 (13.3)	5 (5.8)	13 (15.9)	11 (14.3)	7 (10.0)
	15	Practice	Consultation hours	447 (446/5)	10 (2.2)	1 (1.2)	3 (3.8)	1 (1.3)	0 (0.0)	2 (2.8)	3 (4.8)
	16	Practice	Entertainment	423 (423/12)	15 (3.6)	1 (1.3)	7 (8.6)	2 (2.8)	2 (3.3)	3 (4.4)	0 (0.0)
	17	Practice	Availability (eg, by telephone and email)	421 (417/16)	13 (3.1)	5 (5.6)	4 (4.9)	1 (1.3)	3 (4.9)	0 (0.0)	0 (0.0)
	18	Practice	Practice equipment	407 (404/40)	14 (3.4)	3 (3.5)	1 (1.3)	2 (2.7)	2 (3.1)	3 (5.4)	3 (6.1)
	19	Practice	Parking spaces	386 (386/7)	34 (8.8)	11 (14.1)	8 (11.6)	5 (6.8)	4 (7.5)	4 (7.1)	2 (3.6)
	20	Practice	Accessibility by public transport	346 (346/0)	8 (2.3)	2 (3.2)	0 (0.0)	3 (5.3)	0 (0.0)	2 (3.8)	1 (2.0)
	21	Practice	Barrier-free access	272 (272/4)	9 (3.3)	3 (5.1)	1 (1.9)	2 (4.0)	1 (2.8)	1 (2.7)	1 (2.8)
	22	Practice	Child-friendly environment	203 (203/3)	6 (3.0)	1 (2.7)	1 (2.7)	2 (5.3)	2 (6.3)	0 (0.0)	0 (0.0)

^a^The rating system on jameda consists of 5 mandatory questions, rated according to the grading system in German schools on a 1-6 scale (1=very good, 2=good, 3=satisfactory, 4=fair, 5=deficient, and 6=insufficient). These relate to Nr. 1-5. A mean score (“overall rating”) is calculated based on the results of these 5 questions. In addition, several optional questions are available for answering. Beyond that, a narrative commentary has to be given for every rating.

Table 4 presents the three categories (ie, physician, office staff, and practice) and all 29 corresponding topics that could be derived from the analysis of the 600 narrative comments, as well as from corresponding physician responses. The 600 narrative comments contained 1246 topics, of which most were related to physician (73.76%, 919/1246); in addition, 214 (17.17%, 214/1246) narrative comments contained information about the office staff, and 113 (9.07%, 113/1246) about the practice, respectively. Overall, almost one-third of commented topics were responded to by the physicians (30.66%, 382/1246). Thereby, the response rate varied between 20.6% (44/214) for office staff-related comments and 33.1% (304/919) for physician-related comments, respectively. As displayed, a recommendation for or against consulting a particular physician was given in slightly more than one-third of all narrative comments (35.2%, 211/600), which were answered by approximately every ninth physician (11.9%, 25/211). The second most frequently mentioned topic was an assessment of the professional competence of the physician (28.5%, 171/600). Here, approximately every fifth physician responded to those narrative comment elements (19.9%, 34/177). Higher response rates for more frequently mentioned topics were determined when patients wrote about their medical history; here, almost 4 out of 5 physicians (77.4%, 72/93) responded to the patients’ narrative. Similarly, narrative comments that contained information about treatment costs were answered by 69.4% (29/43) of all physicians.

**Table 4:** An overview of the content of narrative comments, ratings and physician responses on jameda for a randomly selected sample of 600 Web-based ratings (2015), equally distributed among the six overall rating result categories (ie, 100 ratings each).

Number	Category	Topic	Appearances in narrative comments, n (%)	Physician responses, n (%)	Physician response rate per overall rating result (%)					
					Very good	Good	Satisfactory	Fair	Deficient	Insufficient
1	Physician	Recommendation of the physician	211 (35.2)	25 (11.9)	1 (2.4)	2 (10.0)	5 (21.7)	5 (16.7)	6 (12.8)	6 (12.0)
2	Physician	Professional competence	171 (28.5)	34 (19.9)	4 (6.2)	5 (13.2)	7 (24.1)	3 (17.7)	8 (61.5)	7 (77.8)
3	Physician	Overall assessment	121 (20.2)	13 (10.7)	3 (10.3)	1 (4.0)	4 (15.4)	3 (21.4)	1 (6.7)	1 (8.3)
4	Physician	Patient history	93 (15.5)	72 (77.4)	2 (11.1)	5 (100.0)	12 (100.0)	21 (100.0)	19 (100.0)	13 (72.2)
5	Physician	Revenue orientation	73 (12.2)	32 (43.8)	0 (0.0)	3 (33.3)	4 (30.8)	12 (57.1)	4 (36.4)	9 (56.3)
6	Physician	Patient involvement	58 (9.7)	25 (43.1)	1 (10.0)	1 (9.1)	1 (50.0)	10 (83.3)	3 (27.3)	9 (75.0)
7	Physician	Atmosphere	50 (8.3)	20 (40.0)	7 (33.3)	2 (25.0)	2 (33.3)	3 (50.0)	5 (83.3)	1 (33.3)
8	Physician	Treatment cost	43 (7.2)	29 (67.4)	N/A	3 (60.0)	8 (61.5)	6 (54.6)	4 (66.7)	8 (100.0)
9	Physician	SHI^a^-PHI^b^-differentiation	41 (6.8)	25 (61.0)	4 (44.4)	7 (70.0)	1 (25.0)	5 (83.3)	3 (60.0)	5 (71.4)
10	Physician	Being taken seriously	30 (5.0)	5 (16.7)	0 (0.0)	0 (0.0)	2 (100.0)	2 (33.3)	0 (0.0)	1 (14.3)
11	Physician	Communication	16 (2.7)	14 (87.5)	N/A	4 (80.0)	3 (100.0)	1 (100.0)	4 (66.7)	2 (100.0)
12	Physician	Cooperation with medical specialists	7 (1.2)	5 (71.4)	2 (66.7)	0 (0.0)	N/A	2 (100.0)	N/A	1 (100.0)
13	Physician	Privacy	3 (0.5)	3 (100.0)	N/A	N/A	1 (100.0)	N/A	N/A	2 (100.0)
14	Physician	Additional information or advertisement	2 (0.3)	2 (100.0)	N/A	N/A	1 (100.0)	1 (100.0)	N/A	N/A
15	Office staff	Friendliness of the office staff	155 (25.8)	31 (20.0)	4 (10.3)	6 (14.0)	4 (18.2)	5 (27.8)	6 (35.3)	6 (37.5)
16	Office staff	Overall assessment	28 (4.7)	7 (25.0)	2 (33.3)	2 (33.3)	2 (25.0)	0 (0.0)	0 (0.0)	1 (50.0)
17	Office staff	Information and advice	9 (1.5)	2 (22.2)	1 (33.3)	0 (0.0)	0 (0.0)	0 (0.0)	1 (100.0)	0 (0.0)
18	Office staff	Privacy	8 (1.3)	4 (50.0)	0 (0.0)	1 (100.0)	2 (50.0)	1 (100.0)	N/A	N/A
19	Office staff	Recommendation	5 (0.8)	0 (0.0)	0 (0.0)	0 (0.0)	N/A	N/A	N/A	N/A
20	Office staff	Atmosphere	4 (0.7)	0 (0.0)	0 (0.0)	N/A	N/A	N/A	N/A	N/A
21	Office staff	Time spent with the patient	4 (0.7)	0 (0.0)	N/A	0 (0.0)	0 (0.0)	N/A	N/A	N/A
22	Office staff	Trust	1 (0.2)	0 (0.0)	0 (0.0)	N/A	N/A	N/A	N/A	N/A
23	Practice	Office organization	38 (6.3)	27 (71.1)	2 (50.0)	7 (77.8)	5 (71.4)	4 (50.0)	4 (100.0)	5 (83.3)
24	Practice	Atmosphere	26 (4.3)	5 (19.2)	4 (33.3)	0 (0.0)	0 (0.0)	0 (0.0)	0 (0.0)	1 (33.3)
25	Practice	Overall assessment	26 (4.3)	0 (0.0)	0 (0.0)	0 (0.0)	0 (0.0)	N/A	0 (0.0)	0 (0.0)
26	Practice	Recommendation	17 (2.8)	0 (0.0)	0 (0.0)	0 (0.0)	0 (0.0)	N/A	N/A	0 (0.0)
27	Practice	Privacy	4 (0.7)	1 (25.0)	N/A	1 (50.0)	N/A	0 (0.0)	N/A	0 (0.0)
28	Practice	SHI-PHI-differentiation in practice equipment	1 (0.2)	0 (0.0)	N/A	N/A	N/A	0 (0.0)	N/A	N/A
29	Practice	Connection to medical infrastructure	1 (0.2)	1 (100.0)	0 (0.0)	N/A	N/A	N/A	N/A	N/A

^a^SHI: statutory health insurance.

^b^PHI: private health insurance (eg, different waiting rooms and more service comfort for privately insured patients).

## Discussion

### Principal Findings

Physician-rating websites (PRWs) may lead to quality improvements in case they enable and establish a peer-to-peer communication between patients and physicians [19]. Whereas most research has addressed the perspective of rating patients (mentioned previously), less research has focused on the perspective of physicians who are being rated on PRWs [25]. So far, we know little about whether and how physicians respond on the Web to patient ratings. Therefore, the aim of this study was to describe trends in physicians’ Web-based responses to patient ratings over time, to identify what physician characteristics influence Web-based responses, and to examine the topics physicians are likely to respond to. To the best of our knowledge, this is the first study adding knowledge in this regard by presenting the results of a comprehensive analysis based on patient ratings and physician responses displayed on the German PRW, jameda, from 2010 to 2015. As a result, we could show that physicians have responded to less than 2% of all Web-based ratings (1.58%, 16,640/1,052,347). Moreover, less than 3% of all rated physicians have responded on the Web to patient ratings (2.93%, 4187/142,948). Those numbers demonstrate that a Web-based peer-to-peer communication between patients and physicians on such platforms has not been reached [11]. In contrast, further steps seem to be necessary to both enable and further establish such a communication between patients and physicians.

Several requirements need to be satisfied in order to achieve a peer-to-peer communication system between patients and physicians on such websites [1,32-35]. To the best of our knowledge, no such requirements have been discussed in the literature. However, we discuss some requirements as an initial step in the following. First, PRWs must provide the infrastructure for such a dialogue among users [19,32-34]. The results presented in this paper were based on Web-based ratings and corresponding physician responses from the leading German PRW, jameda. However, it is important to mention that jameda is currently the only PRW on which physicians communicate on the Web with patients in a more or less significant manner (ie, in 2015, physicians still responded to less than 2% of all Web-based ratings). To get a more in-depth understanding of the opportunity and current numbers of physician responses to Web-based ratings, we analyzed the 12 most important PRWs in Germany (not presented here in detail). On 5 of those, physicians do not even have the opportunity to respond on the Web to patient ratings. The remaining 6 PRWs have incorporated this option, but we did not find a single physician response here for a randomly selected sample of 400 orthopedic doctors across Germany on any PRW. This additional analysis demonstrates the need to enhance the options for physicians to comment on their patient ratings by providing the relevant infrastructure on those rating websites.

It also seems important that physicians are made aware of the existence of PRWs and make good use of them [4,19]. As shown recently for a sample of 828 physicians affiliated with one of four hospitals in a large accountable care organization in eastern Massachusetts, 53% of those surveyed reported visiting a PRW at least once. Here, a decreasing age, having ambulatory clinical time, and practicing in a surgical specialty or obstetrics or gynecology was associated with visiting a website [36]. Another study from Germany has demonstrated that 67.08% of a survey sample of 2360 outpatient physicians has stated that they use PRWs at least once per month. Only a minority of those who are aware of PRWs stated that they never use them (5.81%, 137/2360) [4]. Even though those numbers appear to confirm the awareness of PRWs among physicians, they are likely to overestimate the real level awareness of physicians in the outpatient sector in both countries. For example, the German sample comprised of health care providers who have either subscribed to a monthly newsletter on jameda or booked a jameda service product. This means that physicians who are less interested in Web-based topics (eg, those without a practice website), or PRWs in general, are less likely to be represented by those results [4]. Nevertheless, the numbers in general seem to indicate that not only patients [3,7,9] but also physicians have become more aware of the PRW movement.

Furthermore, it seems important to gain a better understanding of the purposes for which physicians use PRWs and respond to Web-based ratings [4,36]. So far, little research has addressed those questions. What we know from one German study is that the main reason for using PRWs is to read Web-based ratings for their own practice; here, almost 9 out of 10 physicians (87.08%, 2055/2360) confirmed this to be the main driver for using PRWs [4]. Other important reasons given were to read the ratings of other physicians because of interest (48.69%, 1149/2360) and for marketing purposes (33.26%, 785/2360). Only slightly more than every fourth physician stated they comment on their own ratings (27.80%, 656/2360), confirming the low numbers of Web-based responses to Web-based ratings from the study presented in this paper.

In this context, we could show that 142,948 physicians have been rated on jameda over the 6-year study period from 2010 to 2015. Compared with the overall number of physicians in the outpatient sector (N=157,720 [37]) and dentists (N=52,295 [38]) in Germany in 2015, this means that almost 7 out of 10 physicians have been rated so far (68.07%, 142,948/210,015). The result from this study, that is, that only 3% (4187/142,948) of all rated physicians have responded on the Web to their patient ratings and from our additional analysis for a randomly selected sample of 400 orthopedic doctors across Germany on 6 further German PRWs (see above), emphasizes the need to encourage more physicians to respond to Web-based ratings if we want to establish peer-to-peer communication among users on such platforms [11]. Therefore, PRW providers should take action to foster a more frequent communication process between the patient and the rated physician. This could be realized by the providers of PRWs by taking over a more active role as a moderator between the patient and the physician [19]. For example, PRWs providers should inform rated physicians on a regular basis (eg, monthly) about Web-based patient ratings and enable them to respond to those ratings in an easy manner. Only then could a feedback loop be generated between patients and providers that would create value for both patients and providers.

So far, we know little about why physicians respond or do not respond to Web-based patient ratings. Our analysis has demonstrated that most responses were related to ratings in the middle or lower range of the rating scale. One likely reason is that physicians try to express their point-of-view and explain what consequences the rating will have on the daily practice. For example, Hardey states one example of how a hospital responded to one patient who complained about the friendliness of one doctor and the incomprehensibility of another doctors’ explanation. Referring to this comment, the hospital responded: “Thank you very much for your kind comments particularly regarding Brendan. We have forwarded your comments onto him. However, we were very sorry to read of your experience of the communication with some of our medical staff and we have raised this with the clinical lead of Orthopaedics and A&E to raise with the medical teams. We are glad however that your overall experience was good and the nursing staff supported you”[32]. Such a mechanism might increase the usefulness for both patients and physicians since it becomes possible to understand the concern and the reason for the positive or negative evaluation, as well as the perspective of the rated physician. However, physicians might try to learn from the patient comments in the first place so as to avoid negative patient reviews. In this regard, a comprehensive meta-analysis by de Matos and colleagues has shown that services providers (such as physicians and hospitals) should make every effort to provide services correctly on the first time, rather than permitting failures and then trying to respond with superior recovery [39]. Nevertheless, we still know very little about the motivation of physicians to respond or not respond to Web-based patient ratings. Future research should elaborate on this issue more in detail.

### Limitations

There are some limitations that must be taken into account when interpreting the results of this investigation. First, we analyzed the frequency and content of patient ratings and corresponding physician responses from only one rating website. Although jameda has been shown to be the most frequently used German PRW [4], it is possible that the analysis of other PRWs would have resulted in other findings. However, as stated above, other PRWs are very likely to contain a far lower number of physician responses to Web-based ratings. Second, the quantitative content analysis contained 600 narrative comments that were equally distributed among the six overall rating result categories (ie, 100 ratings each). This means that those results are not likely to represent the real distribution of comments on PRWs. Finally, we did not discuss the level of disagreement between the patient rating and the physician response.

### Conclusions

So far, only a minority of physicians have taken the chance to respond on the Web to patient ratings on the leading German PRW, jameda. This demonstrates that the goal of establishing a Web-based peer-to-peer communication between patients and physicians on such platforms has not been reached [11]. This is likely because of (1) the still-low awareness of physicians of PRWs, (2) the fact that only few PRWs provide the infrastructure for physicians to respond on the Web to patient ratings, and (3) the lack of an active moderator to foster peer-to-peer communication between the patient and the physician. If we want a feedback loop to be generated between patients and health care providers that creates value for both the patients and the providers, health policy makers should implement measures to encourage physicians to respond on the Web to patient ratings. Further research is needed to learn more about the motivation of physicians to respond or not respond to Web-based patient ratings.

## Abbreviations

PRW: physician-rating website
SD: standard deviation
